# The Polarization-Index: A Simple Calculation to Distinguish Polarized From Non-polarized Training Intensity Distributions

**DOI:** 10.3389/fphys.2019.00707

**Published:** 2019-06-12

**Authors:** Gunnar Treff, Kay Winkert, Mahdi Sareban, Jürgen M. Steinacker, Billy Sperlich

**Affiliations:** ^1^Division of Sports and Rehabilitation Medicine, Ulm University Hospital, Ulm, Germany; ^2^Institute of Sports Medicine, Prevention and Rehabilitation, Paracelsus Medical University, Salzburg, Austria; ^3^Integrative and Experimental Exercise Science and Training, Institute of Sport Science, University of Würzburg, Würzburg, Germany

**Keywords:** high-intensity training, high-performance sports, lactate threshold training, endurance training, elite

## Abstract

The training intensity distribution (TID) of endurance athletes has retrieved substantial scientific interest since it reflects a vital component of training prescription: (i) the intensity of exercise and its distribution over time are essential components for adaptation to endurance training and (ii) the training volume (at least for most endurance disciplines) is already near or at maximum, so optimization of training procedures including TID have become paramount for success. This paper aims to elaborate the polarization-index (PI) which is calculated as log_10_(Zone 1/Zone 2^∗^Zone 3^∗^100), where Zones 1–3 refer to aggregated volume (time or distance) spent with low, mid, or high intensity training. PI allows to distinguish between non-polarized and polarized TID using a cut-off > 2.00 a.U. and to quantify the level of a polarized TID. Within this hypothesis paper, examples from the literature illustrating the usefulness of PI-calculation are discussed as well as its limitations. Further it is elucidated how the PI may contribute to a more precise definition of TID descriptors.

## Introduction

The training intensity distribution (TID) of endurance athletes has become an important component of training prescription since (i) the intensity of exercise and its distribution over time are essential components of adaptation to endurance training and (ii) the training volume (at least for most disciplines) is already near or at maximum. Therefore, several optimization procedures have gained scientific interest, including the manipulation of TID, and other components of exercise prescription including exercise duration, volume, frequency, or mode.

To quantify TID, the intensity of exercise is commonly defined according to physiological thresholds and distributed into an “intensity zone-model,” of which a three-zone model is predominantly employed for scientific evaluation. Briefly, the intensity of Zone 1 incorporates low-intensity exercise greater than or equal to 50% of maximal oxygen uptake (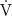
O_2 max_) and lower than the intensity corresponding to the first lactate or ventilatory threshold. Exercise prescription in Zone 1 is often termed “basic-endurance” or “low-intensity” exercise. The first and second lactate or ventilatory thresholds define the lower and upper limits of Zone 2, an exercise intensity that is often termed “threshold intensity,” or “lactate threshold training.” Zone 3 is usually defined as an exercise intensity greater than the second lactate or ventilatory threshold and established as high-intensity interval training near or at maximum 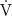
O_2 max_. These training intensities may also be defined by other variables based on blood lactate concentration, percentage of maximal heart rate or 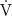
O_2 max_, or subjective ratings like the “Session-RPE”. For details see Seiler and Kjerland (2006), Seiler (2010). However, the physiological transitions between training intensities are fluent and the targeted adaptions depend on multiple factors including training volume, TID, health-, and training-status. For detailed reviews see Gibala et al. (2012), Milanović et al. (2015), and MacInnis and Gibala (2016).

Among several TID patterns, four main distributions have been reported and investigated so far, namely the “polarized”-, “high-intensity”-, “pyramidal”-, and “lactate threshold”-TID, which are based on previous definitions (Seiler and Kjerland, 2006; Stöggl and Sperlich, 2015):

• *Polarized TID* consists of elevated percentages of time or distance spent in both high- (Zone 3) and low-intensity exercise (Zone 1) and only a small proportion of training in Zone 2. The polarized TID with its fractions of training volume spent at low-, threshold-, and high-intensity often consists of e.g., 80% of training volume spent in Zone 1, 5% in Zone 2, and 15% in Zone 3 (80-5-15), or 75-5-20, (i.e., 75% within Zone 1, 5% in Zone 2, and 20% in Zone 3), with percentages of Zone 1 greater than Zone 3 *and* Zone 3 always greater than Zone 2.
• *Pyramidal TID* consists of high percentage of training volume spent in Zone 1 and less proportions in Zone 2 and 3. As an example, a pyramidal TID may be quantified as 70-20-10, i.e., 70% within Zone 1, 20% in Zone 2, and 10% in Zone 3.
• *Threshold TID* consists of training volume emphasizing Zone 2. This distribution is frequently established by longer intervals with an intensity between first and second lactate or ventilatory threshold or by continuous exercise intermixed with higher intensities and without a distinct recovery interval. As an example, a threshold TID could be designed as 40-50-10 (i.e., 40% within Zone 1, 50% in Zone 2, and 10% in Zone 3). Notably, a threshold TID, e.g., 50-45-5 (i.e., 50% within Zone 1, 45% in Zone 2, and 5% in Zone 3), may but not necessarily has to be pyramidal (i.e., with decreasing proportions of Zone 2 and Zone 3).
• *High Intensity TID* is a TID with training predominantly performed in Zone 3 and mainly involving interval training. A typical high-intensity TID could be designed as 20-10-70 (i.e., 20% within Zone 1, 10% in Zone 2, and 70% in Zone 3).

**FIGURE 1:**
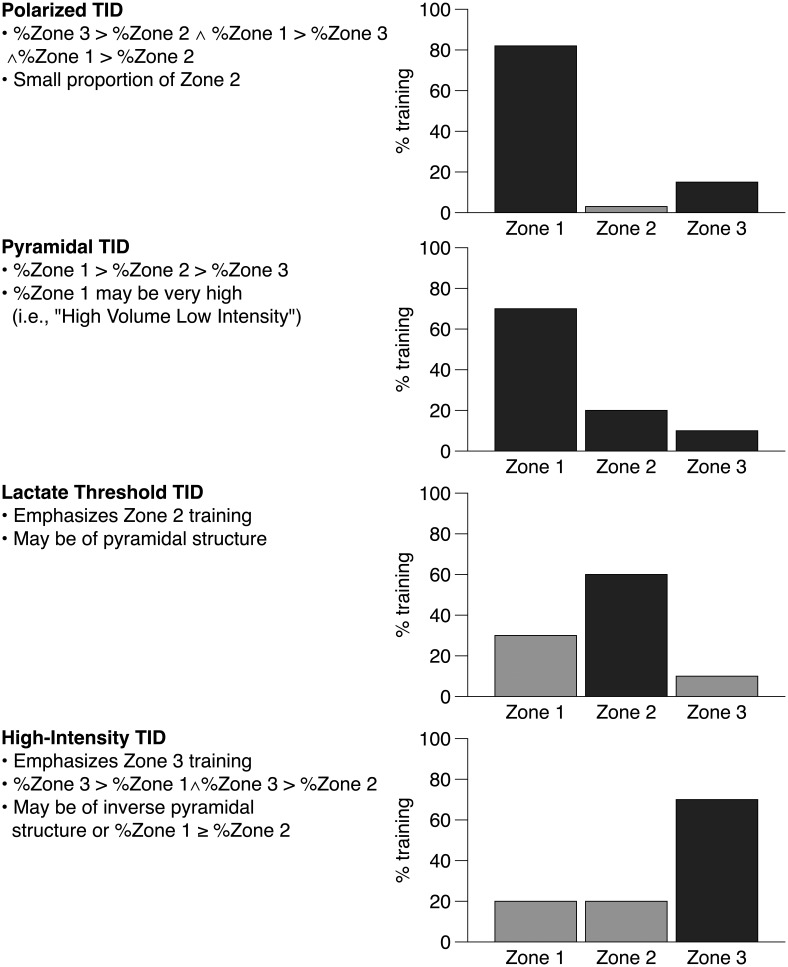
Various training intensity distributions (TID), their schematic proportions, and key characteristics (indicated by black bars). Zones refer to following intensities: Zone 1 (basic endurance), ≥ 50% 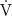
O_2 max_ and ≤ first lactate or ventilatory threshold; Zone 2 (lactate threshold), ≥ first and ≤ second lactate or ventilatory threshold; Zone 3 (high intensity), > second lactate or ventilatory threshold.

Notably, the classification of TIDs to one of the four patterns shown in Figure 1 maybe ambiguous. Especially the term “polarized” differs substantially between publications (Stöggl and Sperlich, 2015; Plews and Laursen, 2017), nevertheless the polarized TID has received increasing scientific interest since retrospective analysis (Seiler and Kjerland, 2006), and prospective randomized-controlled trials have documented equal (Ingham et al., 2008; Treff et al., 2017) or superior gains in endurance performance (Neal et al., 2013; Stöggl and Sperlich, 2014; Tønnessen et al., 2014) when compared to the pyramidal, threshold, or high-intensity TIDs. “Polarized” TID comprises a variety of fractions of Zone 1-3 and is sometimes even used as a descriptor for pyramidal TIDs (Plews and Laursen, 2017) which are clearly characterized by decreasing proportions of Zone 1, 2, and 3, or for TIDs that do not differentiate between Zone 2 and Zone 3 (Fiskerstrand and Seiler, 2004), thereby violating the aforementioned TID classification. Therefore, the definition of polarized vs. other non-polarized TID is often unclear and sometimes misleading.

For this reason, we would like to present an elaborated concept of our previously published polarization-index (PI) (Treff et al., 2017), which is based on the assumption of two necessary conditions for a polarized TID. (i) a polarized *structure*, where Zone 1 > Zone 3 and Zone 3 > Zone 2 (and consequently Zone 1 > Zone 2) and (ii) a relatively small proportion of Zone 2. The PI aims to distinguish between polarized and non-polarized TID and to quantify the level of a polarized TID. Further, we aim to highlight the PI’s usefulness and limitations, thereby contributing to a more precise TID terminology within the scientific literature. Based on studies published between 2009 and 2018 and reported in our previous paper, we want to highlight examples illustrating why we believe the polarization index may be a valuable tool for practical and scientific purposes.

## Calculation of the Polarization-Index

The formula for calculation of the PI is based on a three-zone TID-model:

(1) $$Polarization-index(a.U.)=\log{\;}_{10}(Zone\text{ 1/}Zone\text{ 2}\times Zone\;3*100)$$

where Zone is the fraction (given percentage/100) of the training volume in Zone 1, 2, and 3.

The PI increases if a high ratio of Zone 1 to Zone 2 is combined with a high percentage of training in Zone 3. The log-transformation of the raw-data establishes a quasi linear function.

If Zone 2 = 0, Eq. 2 avoids zero in the denominator:

(2) $$Polarization-index(a.U.)=\log{\;}_{10}(Zone\text{ 1/}0.01\;\times Zone\;3-0.01*100)$$

If Zone 3 = 0, PI is zero per definition.

If Zone 3 > Zone 1 the PI is not valid and must not be calculated (please see discussion for details below).

If PI > 2.00 a.U., the TID is defined as “polarized,” with increasing values indicating a higher level of polarization. If PI is ≤ 2.00 a.U., the TID is defined as non-polarized.

## Justification of the Pi-Concept

For a polarized TID we assume (i) a polarized structure and (ii) a relatively small proportion of Zone 2.

**FIGURE 2:**
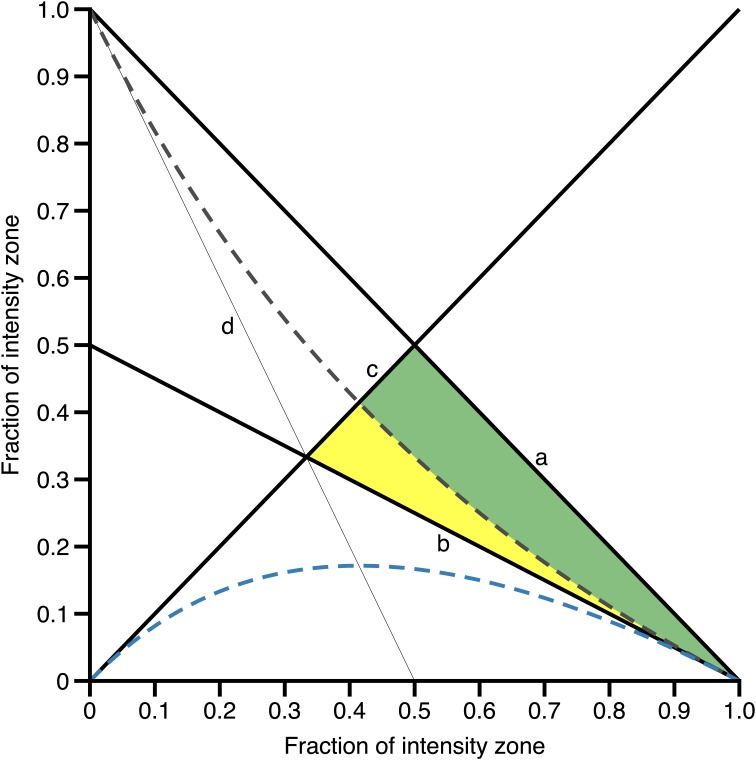
Limits and conditions of the polarization index. Line a indicates the upper limit of Zone 3 for a given (x-axis) fraction of Zone 1 (condition a: Zone 1 + Zone 2 + Zone 3 = 1), line b indicates the lower limit of Zone 3 for a given fraction of Zone 1 (condition b: Zone 3 > Zone 2), line c indicates the upper limit of Zone 3 for a given fraction of Zone 1 (condition c: Zone 1 > Zone 3), and line d indicates the lower limit of Zone 3 for a given fraction of Zone 1 (condition d: Zone 1 > Zone 2). The colored areas represent the range of values where conditions a-d are fulfilled. The gray broken line indicates the limit of Zone 3 for a given fraction of Zone 1 resulting in a polarization-index of 2.00 a.U., i.e., if Zone 3 is higher or lower, polarization-index will result in values higher (green area) or lower than 2.00 a.U. (yellow area), respectively. The broken blue line indicates the upper limit of Zone 2 for a given fraction of Zone 1 to allow for a polarization-index ≥ 2.00 a.U.

Ad (i) For the polarized structure, we agree on the following necessary conditions: a: Zone 1 + Zone 2 + Zone 3 = 1, b: Zone 3 > Zone 2, c: Zone 1 > Zone 3, and d: Zone 1 > Zone 2. Figure 2 visualizes these conditions over the range 0–1 (or 0 – 100%) and the colored areas represent the range of fractions where all of these conditions are fulfilled.

Due to these conditions, a PI > 2.00 a.U. is inevitably associated with a polarized structure, because if Zone 2 equals Zone 3 (a TID which does precisely not stand for a polarized structure), the result of Zone 1/Zone 2 × Zone 3 will equal the value of Zone 1, since Zone 2 and Zone 3 will shorten each other, e.g., 0.8/0.2 × 0.2 ^∗^100 = 80. Zone 1 approaches the maximal value of 100%, therefore the raw, i.e., not log-transformed PI will approach a value of 100. Since log_10_(100) equals 2.00, the PI approaches 2.00 a.U. and cannot exceed a value > 2.00 a.U. if Zone 2 equals Zone 3. Consequently, the percentage of Zone 3 must be higher than Zone 2 to result in a PI > 2.00 a.U. Or, vice versa: If PI > 2.00 a.U., the necessary condition *Zone1 > Zone3 ∧ Zone 3 > Zone 2* will be met in each case for Z1 ≤ 100% (Figure 2, green area).

Ad (ii) At the same time the 2.00-threshold can be employed to identify the fulfillment of the second necessary condition for a polarized TID, i.e., comprising of “a relatively small” proportion of Zone 2: If, for example, the percentage of Zone 1 is as low as 60%, a TID of 60-19-21 will result in a PI of 1.82 a.U., thereby clearly below the cut-off and violating the definition of a polarized TID, even though Zone 1 > Zone 3 ∧ Zone 3 > Zone 2 (i.e., the first necessary condition for a polarized TID is *true)*. However, if Zone 2 is lower than ∼ 15% in this example, the PI will be > 2.00 a.U. and consequently, PI calculates 2.05 a.U. in a 60-14-26 distribution.

Of note, with higher percentages of Zone 1 training (e.g., 80%), a Zone 2 percentage lower than 9% will already allow for a polarized TID (e.g., 80-8-12) resulting in a PI of 2.08 a.U. This behavior is visualized by the broken blue line in Figure 2, indicating the upper limit of Zone 2 allowing for a PI ≥ 2.00 a.U. and the approximation of the broken lines to line b with increasing fractions of Zone 1.

The cut-off > 2.00 a.U. is therefore not arbitrary and clearly providing a benchmark for a polarized structure and an objective (but not physiological) definition of “relatively small” percentages of Zone 2 training in polarized TIDs.

## Application of the Polarization Index in Training Intensity Studies

Table 1 shows several original investigations published between 2009 and 2018 which were discussed in one of our previous papers (Treff et al., 2017), their PI and the employed descriptor.

**Table 1:** Selected studies in the area training intensity distribution, percentages of three training intensity zones, the resulting polarization-index, and their classification according to the polarization-index and the original publication.

	Authors	Time or distance spent in			Polarization-Index (a.U.)	Classification according to	
		Zone 1 (%)	Zone 2 (%)	Zone 3 (%)		Polarization-Index	Authors
1	Neal et al., 2013	80.0	0.0	20.0	3.18	Polarized	Polarized
2	Ingham et al., 2008	72.0	0.0	28.0	3.29	Polarized	Polarized
3	Stöggl and Sperlich, 2014	68.0	6.0	26.0	2.47	Polarized	Polarized
4	Bourgois et al., 2013	93.1	2.3	4.6	2.27	**Polarized**	**Not classified**
5	Treff et al., 2017	93.0	1.0	6.0	2.75	Polarized	Polarized
6	Neal et al., 2013	57.0	43.0	0.0	0.00	Non-polarized	Non-polarized (LT)
7	Stöggl and Sperlich, 2014	46.0	54.0	0.0	0.00	Non-polarized	Non-polarized (LT)
8	Plews and Laursen, 2017	67.3	30.2	2.5	0.75	Non-polarized	Non-polarized (pyramidal)
9	Stöggl and Sperlich, 2014	83.0	16.0	1.0	0.71	Non-polarized	Non-polarized (HVLT Int)
10	Plews and Laursen, 2017	80.4	17.9	1.8	0.91	**Non-polarized**	**Polarized**
11	Plews et al., 2014	77.3	16.9	5.8	1.42	**Non-polarized**	**Polarized**
12	Treff et al., 2017	94.0	4.0	2.0	1.67	Non-polarized	Non-polarized (pyramidal)
13	Guellich et al., 2009	95.0	3.0	2.0	1.80	Non-polarized	Non-polarized
14	Carnes and Mahoney, 2018	74.0	11.0	15.0	2.00	**Non-polarized**	**Polarized**
15	Stöggl and Sperlich, 2014	43.0	0.0	57.0	n.a.	–	HIT

*LT, lactate threshold training; Hi HVLT, high volume low intensity; HIT, high intensity training. Bold letters indicate that classification according to polarization-index differs from classification in the original publication.*

Five studies of Table 1 (Ingham et al., 2008; Bourgois et al., 2013; Neal et al., 2013; Stöggl and Sperlich, 2014; Treff et al., 2017) report polarized TIDs according to the PI and the aforementioned definitions i.e., Zone 1 > Zone 2 ∧ Zone 3 > Zone 2 as well as low percentage of Zone 2. One study (Neal et al., 2013) reports a 80-0-20 polarized TID evident with a high PI of 3.18 a.U. In one study (Ingham et al., 2008) the index is even higher, due to lower and greater fractions of Zones 1 and 3, respectively. In three studies the PI was lower compared to the TID of the aforementioned studies (i.e., the level of “polarization” was lessened), due to the greater percentage of Zone 2 (Bourgois et al., 2013; Stöggl and Sperlich, 2014) or lower absolute percentage spent in Zone 3 (Treff et al., 2017).

In two studies of Table 1 (Neal et al., 2013; Stöggl and Sperlich, 2014) the percentage of training spent in Zone 3 equaled zero. According to the aforementioned definition, the PI amounts to zero and consequently the TID of both studies are not polarized.

In five studies of Table 1 (Guellich et al., 2009; Plews et al., 2014; Stöggl and Sperlich, 2014; Plews and Laursen, 2017; Treff et al., 2017) the PI varied from 0.71 to 1.80 a.U. representing non-polarized TIDs due to a PI ≤ 2.0 a.U. However, the PI varies reasonably according to the respective contributions of Zone 1, Zone 2, and Zone 3. In detail, the PI is very similar in two examples (Stöggl and Sperlich, 2014; Plews and Laursen, 2017) but clearly higher compared to other studies (Plews et al., 2014; Treff et al., 2017) with a similar fraction of Zone 2, but a nearly threefold higher percentage of Zone 3 at the expense of a lower percentage in Zone 1.

Table 1 also illustrates a special TID variant, as the study by Carnes and Mahoney (2018) represents a TID (74-11-15) in which the percentage of Zone 3 is higher compared to Zone 2, indicating a polarized structure, however the fraction of time spent in Zone 2 is considerably high, thereby not fulfilling the second necessary criterion for a polarized TID (Zone 2 being relatively small), which is mirrored by the PI of 2.00 a.U., indicating a non-, but “nearly”-polarized TID.

## Classification and Detailed Quantification of Training Intensity Distributions in the Literature

As already mentioned, the term “polarized” superficially describes the TIDs within retrospective training analysis and prospective experiments. For example, the “polarized” TIDs of the studies summarized in Table 1 (Ingham et al., 2008; Bourgois et al., 2013; Neal et al., 2013; Stöggl and Sperlich, 2014) report substantial differences in the fraction of Zone 3 ranging from 6 to 28%. Taking into account that a large body of evidence has revealed that training emphasizing Zone 3 promotes substantial differences in oxygen transport and utilization (Hickson et al., 1977; Milanović et al., 2015), it seems important to know how “polarized” an experiment was in order to judge the level of adaptation in connection with various TIDs, and to allow better comparisons between groups and studies.

Table 1 also provides examples of studies reporting a polarized TID, even though the percentage of Zone 2 is considerably high (≥17%) and proportions of Zone 1 to 3 continuously decrease, thereby clearly indicating a pyramidal TID (Plews et al., 2014). The borderline TID by Carnes and Mahoney (2018) also claims to have employed a “polarized” TID. In each of these studies, the application of the PI would provide a more precise and objective classification of the respective TIDs.

## Application of the Polarization-Index in Training Monitoring and Analysis

**FIGURE 3:**
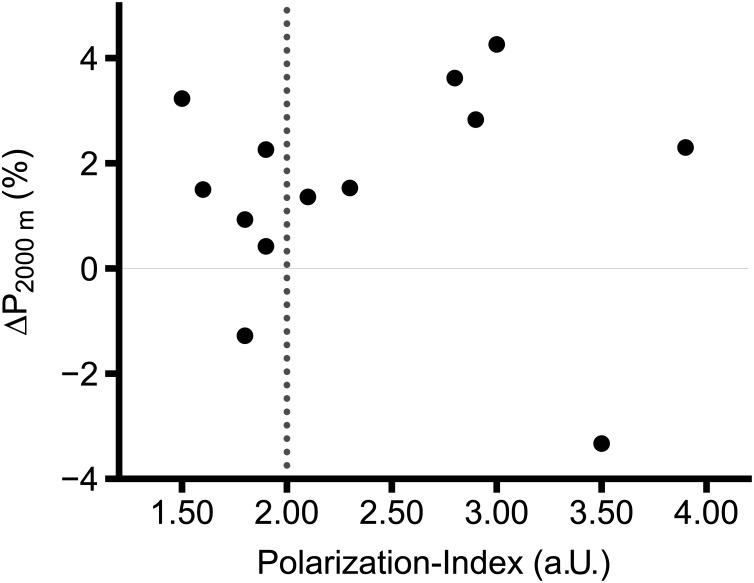
Percentage change of average power in 2000 m rowing ergometer test (P_2000m_) in internationally competing rowers. Vertical dashed line represents the cut-off between non-polarized (≤ 2.00 a.U.) and polarized (> 2.00 a.U.) TIDs. Figure adapted from Treff et al. (2017).

Figure 3 is an example retrieved from a previous paper of our group, illustrating the practical application of the PI in a scientific study simultaneously illustrating the practical application of the PI for training monitoring, and analysis with two groups of rowers performing either a pyramidal (PI = 1.70 a.U.) or a polarized TID (PI = 2.70 a.U.) (Treff et al., 2017). As in similar training studies, the TIDs differed significantly between groups, but at the individual level, the training was quite heterogenous. The PI therefore might allow for a more precise analysis of training outcomes taking into account the actual individual TID. Further, the PI can easily be integrated into standard training monitoring software.

However, some rules should be followed when interpreting the PI:

As shown before, very small differences between Zone 2 and Zone 3 will allow for a PI ≥ 2.00 a.U. if percentage of Zone 1 is high. Therefore, the PI is practically useful within reasonable and accepted limits for Zone 1 in polarized TIDs, being approximately 70–90% (Stöggl and Sperlich, 2014). Also, a given TID with, for example, 15 h/week training will affect performance differently to the same TID with a volume of, for example, 25 h/week. Therefore, interpretation of changes in performance in relation to a given TID (as illustrated in Figure 3) is only justified when other important variables of training (e.g., training volume, frequency, or training modalities) are clamped, or are at least similar between subjects or within subject. In this case, we would like to emphasize that the PI as an algorithm assists in discriminating between various TIDs, but we discourage the interpretation as a surrogate for training load. For example, a PI of 2.00 a.U. may result out of two substantially different TIDs, e.g., 90-5-5 and 74-13-13. As explained above, and from a biological and empirical perspective, these two training regimes, unlike TID, will result in different central and peripheral adaptations and will affect performance differently, even if applied in a theoretically perfect model, i.e., two identical subjects. It is also worth to mention, that the quality of training data and a reliable and valid allocation of the intensities is crucial for analysis. Finally, successful training is not only a quantitatively but also strongly qualitatively determined intervention and not reflected by the PI or other quantitative training variables.

## Limitations

Despite the practical usefulness, the PI has some limitations that warrant a brief discussion. Even though the PI provides an objective cut-off to distinguish polarized from non-polarized distributions, it does not allow the differentiation of sub-types of the non-polarized TID structures (for example, lactate-threshold vs. high-intensity TID) and values between 0 and 2.00 must not be interpreted in terms of more or less polarized distributions. Furthermore, from a theoretical perspective, it appears inappropriate to replace 0.00% in Zone 2 by e.g., 1.00% to avoid zero in the denominator (Eq. 2). However, from a practical perspective, it is virtually impossible to achieve high intensities (i.e., Zone 3), without some fraction of time spent in Zone 2. Therefore, this limitation appears to be practically irrelevant. Finally, the PI is a statistical measure that increases data density and thereby - like every index - leads to a loss of detailed information.

## Conclusion

The application of the PI represents an algorithm to distinguish distinctively between polarized and non-polarized TIDs and to judge the level of polarization. As shown, the PI has the potential to reduce the ambiguity regarding the classification of TIDs in the current literature and is easily applicable in any training monitoring software. Since the PI also allows intra-individual assessment of polarization, we aim to stimulate researchers to re-evaluate their existing data retrospectively in order to investigate the response or non-response to various TIDs. In addition, we encourage future training experiments to state the level of polarization allowing for more detailed comparison between studies.

## Author Contributions

GT drafted the manuscript. GT, KW, MS, JS, and BS contributed substantially to the manuscript.

## Conflict of Interest Statement

The authors declare that the research was conducted in the absence of any commercial or financial relationships that could be construed as a potential conflict of interest.
